# Development of Real-Time TDDFT Program with **k**-Point Sampling and DFT + *U* in a Gaussian and Plane Waves Framework

**DOI:** 10.1021/acs.jctc.4c01515

**Published:** 2025-02-08

**Authors:** Kota Hanasaki, Sandra Luber

**Affiliations:** Department of Chemistry, University of Zurich, 8057 Zurich, Switzerland

## Abstract

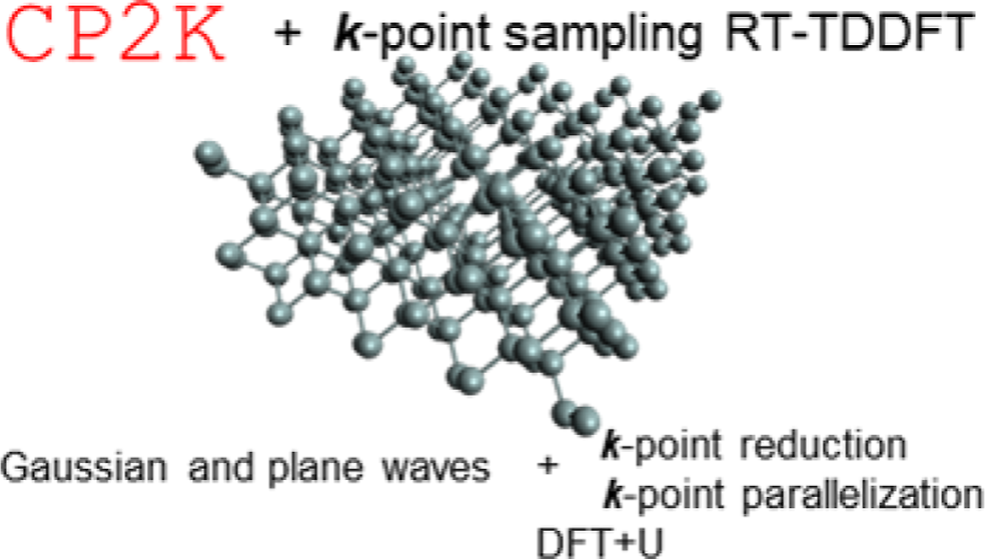

We developed a **k**-point sampling real-time TDDFT (RT-TDDFT) program within the Gaussian and plane waves (GPW) framework of the CP2K software suite. In addition to standard real-time propagation of time-dependent Kohn–Sham orbitals, we make use of symmetry-based **k**-point reduction and **k**-point parallelization schemes so that our RT-TDDFT program in the GPW framework is feasible for practical large-scale calculations. We also implemented DFT + *U* as a relevant extension for real-time simulations of systems with strong electron correlations. In particular, we extended the “tensorial” subspace representation approach for DFT + *U*, following the formulation in [Chai, Z., et al. *J. Chem. Theory Comput.*, **2024,***20,* 8984], to **k**-point sampling RT-TDDFT. Our extension, which is, to our knowledge, the first application of the “tensorial” subspace representation approach to **k**-point sampling RT-TDDFT, is found to be robust and efficient with small additional costs owing to the locality of Gaussian basis functions, indicating that it is a promising approach to RT-TDDFT + *U* for solids. We show details of our implementation in CP2K and the results of our benchmark calculations.

## 1 Introduction

Rapid progress in experimental techniques has been realizing control and observation of excited-state dynamics of molecules and solids.^1−3^ One of the most interesting applications of those techniques is the optical manipulation of electromagnetic properties of materials, which is closely related to potential device applications.^4,5^ Among many ab initio calculation techniques in this research field, the time-dependent density functional theory (TDDFT)^6^ is among the most useful tools because of its good balance between computational cost and accuracy.

We report our development of a real-time TDDFT (RT-TDDFT) code for periodic boundary conditions with **k**-point sampling in the CP2K program suite.^7^ The existing program was extended to include **k**-point sampling since it is indispensable in the excited-state calculations of solids for the reproduction of band structures and for the efficiency of calculations.

Based on the Runge-Gross theorem,^6^ RT-TDDFT has been developed as a real-time approach to excited-state dynamics in molecules and solids. One of the most remarkable applications of RT-TDDFT is real-time simulations of general nonperturbative dynamics.^8−12^ Another important application is the calculation of excitation spectra in perturbative external fields. The real-time approach to spectroscopic analysis in the context of modern electronic structure theory has been pioneered by Yabana and Bertsch^13^ and has been further developed by a number of authors. The application of RT-TDDFT now extends to solids,^14^ magnetic spectra,^15^ resonance Raman spectroscopy,^16^ Raman optical activity,^17^ etc. The accuracy of RT-TDDFT in spectroscopic calculations is known to be approximately the same as that of the linear-response (LR) TDDFT^18,19^ whereas RT-TDDFT has computational advantages in the calculations of large systems with a high density of states and/or spectra of a wide energy range.^20,21^ Our goal is to develop **k**-point sampling RT-TDDFT as a general-purpose tool, whereas in this paper, as the first step of our development, we focus on spectroscopic applications.

CP2K^7^ is an actively updated electronic structure software package including DFT, Hartree–Fock, the second-order Møller–Plesset perturbation theory, etc. It is equipped with a number of advanced computational techniques including load-balancing and efficient storage of sparse matrices,^22^ which are relevant to realize efficient real-time simulations. Another characteristic of CP2K is that it adopts the mixed Gaussian and plane waves (GPW) scheme,^23,24^ in which orbitals are expanded in Gaussian atomic orbitals. The plane waves are used as an auxiliary basis set for efficient calculation of the Hartree potential.^23,24^ Since this scheme is not popular in existing RT-TDDFT programs for solids,^25−30^ we will show details of our implementation. For a **k**-point sampling RT-TDDFT program to be of practical use, efficient computation schemes for simulations of a large number of **k**-points on parallel computers are indispensable. We therefore developed two most fundamental schemes; symmetry-based **k**-point reduction and **k**-point parallelization. We show how these are implemented in CP2K.

In addition to the standard real-time propagation code, we also implemented DFT + *U*(31,32) as a potentially relevant extension of our RT-TDDFT. In this paper, we only consider the “empirical” formulation of DFT + *U* in which the effective on-site repulsion is set as an input parameter. DFT + *U*,^31^ which was originally developed for the calculations of systems with strong electron correlations, has been recently applied to a wider range of materials to reproduce the band gaps^33^ or d band energy levels.^34^ Its low computational cost and ability to correct excitation energies work favorably in RT-TDDFT calculations.^34,35^ An interesting point is that the procedure of projecting the density matrix onto a set of localized orbitals, which is a critical step in DFT + *U*, is not unique but dependent on the basis set and the implementation of DFT. In our GPW-based RT-TDDFT, we can utilize the locality of the Gaussian basis set. An obvious choice of localized orthogonal orbitals constructed from **k**-adapted Gaussian basis functions is a set of localized Löwdin orbitals,^36^ whereas a recent implementation of the “tensorial” subspace representation^37^ approach in CP2K^38^ (within the Γ-point formulation) suggests the use of the orbitals of isolated atoms. We show that the latter is more robust and suited for RT-TDDFT applications with **k**-point sampling.

This paper is organized as follows; in Section 2, we show the formulation and implementation of our program, in Section 3, we show benchmark calculation results, and Section 4 is devoted to a summary and future perspectives.

## 2 Formulation

We solve the time-dependent Kohn–Sham (TDKS) equation
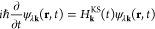
1where ψ_λ**k**_(**r**, *t*) represents a TDKS orbital with Bloch vector^39^**k** and band index λ. TDKS orbitals are expanded as
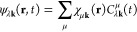
2where *C*_λ**k**_^μ^(*t*) are time-dependent coefficients and χ_μ**k**_(**r**) are **k**-adapted Gaussian atomic orbitals^39^ formally constructed from the μth local Gaussian AO χ_μ_ as
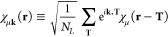
3with *N*_L_ being the number of cells/**k**-points in the simulation and **T** representing lattice translation vectors. During the real-time propagation, we work on the time-dependent coefficients *C*_λ**k**_(*t*) and the basis functions χ_μ**k**_(**r**) do not explicitly appear in calculations. Matrix elements of a given lattice periodic operator are computed from the real-space expression as
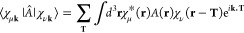
4for a (periodic) potential-type operator *Â*, and
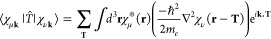
5for the kinetic operator *T̂*, respectively. In numerical calculations, the summation over lattice vectors **T** is truncated at a finite number of neighboring cells. The overlap matrix, for example, is computed from real-space Gaussian overlaps *S*^μν^(**T**) ≡ ∫*d*^3^**r**χ_μ_^*^(**r**)χ_ν_(**r** – **T**) by a numerical Fourier transformation *S*_**k**_^μν^ = ∑_**T**_*S*_μν_(**T**)*e*^*i***k**·**T**^.

### 2.1 Propagator and Propagation Scheme

Equation 1 is integrated with a fixed time step Δ*t* using a multistep Crank–Nicolson (CN) propagator^11,40,41^
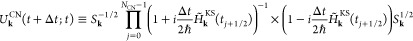
6where *N*_CN_ is the number of substeps, *t*_*j*_ ≡ *t*+(*j*/*N*_CN_)Δ*t* is the *j*th time point, and *S*_**k**_ and  represents the overlap matrix *S*_**k**_^μν^ ≡ ⟨χ_μ**k**_|χ_ν**k**_⟩ and the KS Hamiltonian in the Löwdin representation, , respectively. The KS Hamiltonian  at intermediate time points *t*_*j*_ are linearly interpolated as . We also implemented the enforced time-reversal symmetry (ETRS) propagator^42^
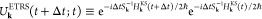
7by extending the existing code in CP2K,^43^ in which multiple algorithms for matrix exponential calculations^44^ are available; the Arnoldi iteration algorithm, Padé approximation, and Taylor expansion. Comparisons of time performance and accuracy among these propagators are shown in Appendix A. We found that there are no significant differences in the time performance for calculations of small unit cells, whereas the best accuracy, estimated by the smallness of the numerical nonorthogonality of TDKS orbitals, was achieved by the CN propagator. We hereafter use the CN propagator with substep division *N*_CN_ = 2.

The TDKS orbitals are propagated keeping the self-consistency between the density matrix and the Kohn–Sham Hamiltonian *H*^KS^. We introduce the time-dependent density matrix *D*_**k**_^μν^(*t*) as
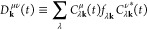
8with *f*_λ**k**_ being the occupation number of TDKS orbital ψ_λ**k**_. At each step of propagation, we require self-consistency of *D*_**k**_^μν^(*t* + Δ*t*) calculated with propagated TDKS orbital coefficients *C*_λ**k**_^μ^(*t* + Δ*t*) and *H*^KS^(*t* + Δ*t*) used in the propagator. Such self-consistency is achieved in an iterative procedure using the direct inversion of iterative space (DIIS)^45^ algorithm as a convergence accelerator.^41^

### 2.2 Reduction of the **k**-Space

In fixed nuclei calculations of crystalline solids, one can reduce the **k** space from the full Brillouin zone (FBZ) to the irreducible BZ (IRBZ). This technique is critically important in large-scale calculations. Since matrices in CP2K are computed in the real-space representation, we need to reproduce the full density matrix to get results equivalent to those in FBZ calculations. We show details of our implementation.^46^

We assume that our target system has crystal symmetry group . Operations in this group are represented as  which consists of a rotation (with possible reflection) α and subsequent translation along the rotation axis **b**, i.e.
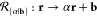
9aand the corresponding operation on the wave function ψ(**r**) is^47^
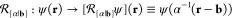
9b

We also introduce notations of atomic coordinate transformation. In general,  maps the *A*th nuclear coordinate in the unit cell at the origin to another nuclear coordinate in the same or a nearby unit cell. We define such an index transformation *f*_{α|**b**}_(*A*) and lattice translation Δ_{α|**b**},*A*_ by
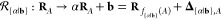
9cwhere we decomposed the transformed coordinate α**R**_*A*_ + **b** to the atomic coordinate at the unit cell at the origin  and the cell coordinate Δ_{α|**b**},*A*_.^48^

By symmetry, KS orbitals satisfy  with a possible phase factor θ, and the occupation number of the orbital ψ_*n*(α**k**)_ equals that of ψ_*n***k**_. The density matrix in the coordinate representation is therefore constructed as
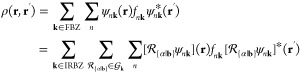
10where  represents a set of symmetry operations that map the IRBZ *k*-point **k** to its distinct equivalent *k*-points in FBZ.

Since the basis functions in CP2K are atomic Gaussian functions with a definite angular momentum around the atomic center, χ_μ_ is characterized by its angular momentum _μ_ and the atomic index *A*_μ_. The coordinate of the *A*_μ_th atom in the unit cell at the origin is denoted as **R**_*A*μ_. It can therefore be shown (see Appendix B) that **k**-adapted Gaussian basis functions rotate as
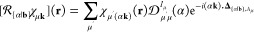
11with  being the rotation matrix for angular momentum . In the RHS of eq 11, index μ′ runs through those of AOs belonging to the same atom and the same angular momentum as χ_μ_. The explicit expression of the rotation matrix  in eq 11 is
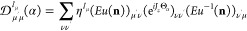
12where *J*_*i*_ (*i* = *x*, *y*, *z*) represents the angular momentum operator/matrix, Θ_α_ is the rotation angle associated with the rotation α, η takes value −1 if α includes reflection and 1 otherwise, *Eu*(**n**) represents the Euler rotation matrix that rotates a unit vector in the *z*-direction to **n**
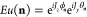
13with θ_**n**_ and ϕ_**n**_ being the polar and azimuthal angles of **n**, respectively.

Hence eq 10 is rewritten as
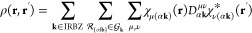
14with *D*_α**k**_^μν^ being the rotated density matrix. To get the orbital rotation eq 11 in a systematic manner, we exploit the atom-based matrix partitioning in CP2K. The partition of a given matrix  consisting of rows of AOs belonging to atom A and columns of AOs belonging to atom B is hereafter referred to as block , where we added an underline to emphasize that it is a matrix block (or, mathematically, a submatrix). We can then calculate the rotated density matrix block-by-block as
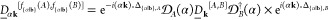
15where  is a block diagonal matrix that consists of  of each angular momentum component in the set of AOs belonging to the *A*th atom.

Although this reconstruction of the density matrix requires additional computation, the total computational time of intermediate to large **k**-point sampling calculations is reduced because the computational time scales approximately linearly with the number of **k**-points. To check the time performance of our implementation, we performed real-time simulations for the optical absorption spectrum (OAS) calculation (see Subsection 3.1 for details) of bulk silicon as a benchmark. The silicon lattice was represented by a cubic 8-atom unit cell with lattice parameter *a* = 5.4306975 Å. We used Goedecker–Teter–Hutter (GTH) pseudopotential,^49^ and double-ζ basis function adapted for the GTH pseudopotential (DZVP-GTH-PADE) implemented in CP2K^50^ as the Gaussian basis set. For all calculations shown in this paper, we applied the Fermi–Dirac smearing with temperature *T* = 300 K. We used the self-interaction corrected (SIC) LDA formulated by Perdew and Zunger^51^ as the *xc* functional. The Brillouin zone was sampled using the Monkhorst–Pack scheme^52^ with 4 × 4 × 4, 6 × 6 × 6, 8 × 8 × 8, 12 × 12 × 12, 16 × 16 × 16, and 20 × 20 × 20 **k**-point meshes. We propagated the TDKS orbitals in a step-function vector potential of strength 0.001 in atomic units, the step size was set Δ*t* = 8 as and we propagated the TDKS orbitals for *N*_step_ = 20 steps (simulation time *T* = 160 as) to get averaged step propagation times.

**Figure 1:**
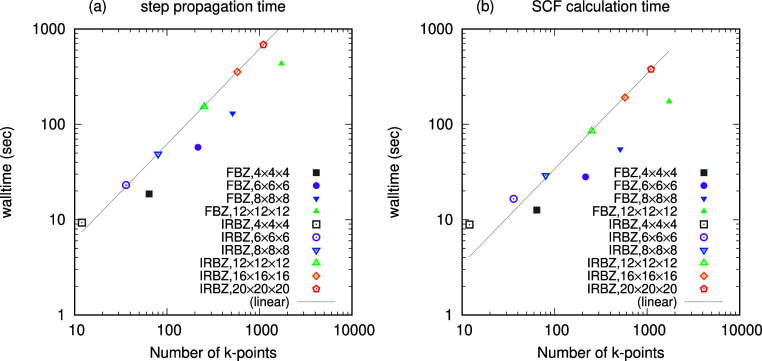
Comparison of (a) averaged single-step propagation time and (b) SCF calculation time in FBZ and IRBZ calculations. Filled (open) symbols show the values for FBZ (IRBZ) calculation with **k**-point mesh indicated in the figure. Thin dotted lines show the linear scaling for reference.

Averaged step-propagation times are shown in Figure 1a. We also show the time for the zeroth step as the approximate ground state self-consistent field (SCF) calculation time in Figure 1b. We find that, in all sets of calculations, the step propagation time and SCF calculation time scale approximately linearly to the number of **k** points. We also find small overheads in the IRBZ step propagation times in comparison to those of FBZ. We attribute these overheads to additional calculations we described above. We can, however, confirm that the computation times using the IRBZ scheme are significantly reduced in comparison to those in an equivalent calculation using the FBZ scheme by checking the computation times indicated in Figure 1 with open (IRBZ) and closed (FBZ) symbols of the same shape.

### 2.3 Parallel Computation

Realistic applications of RT-TDDFT require a large number of simulation steps and dense **k**-point sampling. An efficient parallelization is therefore critical for RT-TDDFT.

In CP2K, there are two types of parallelization available. One is the matrix block parallelization. Many of the relevant matrices appearing in the TDKS equation are stored in the form of distributed block compressed sparse row (DBCSR)^22^ matrices, which are partitioned into atomic blocks as we described in Subsection 2.2. These blocks in real-space matrices are distributed over all message passing interface (MPI) processes. Another type of parallelization is the **k**-point parallelization, where *N*_**k**_**k**-points are divided into *N*_g_ equal-sized **k***-*groups for which *N*_r_ MPI processes are assigned (*N*_g_ is set to be a common divisor of *N*_**k**_ and the total number of MPI processes *N*_p_).

**Figure 2:**
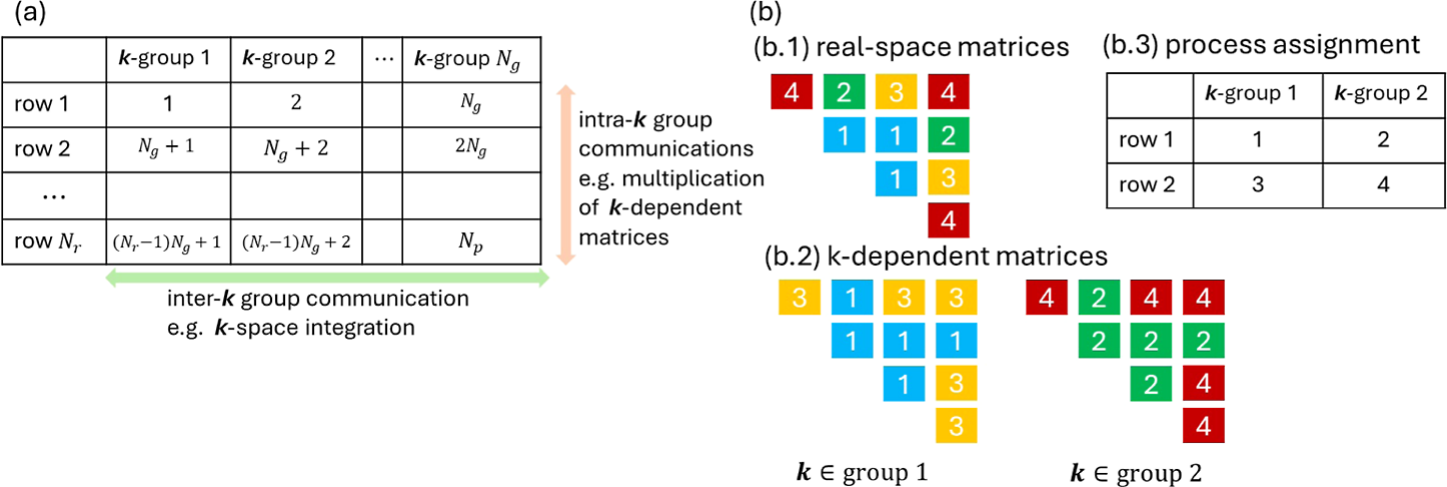
Schematic views of the two-dimensional grid of MPI processes. (a) The arrangement of *N*_p_ MPI processes in *N*_r_ × *N*_g_ grid. (b) The case of *N*_r_ = 2 and *N*_g_ = 2 for an example. Panel (b.3) shows the 2 × 2 grid. Panel (b.1) shows the block assignment of a real-space matrix *A*. The number in each colored box shows the index of the MPI process where the block is stored. The blocks of the Fourier-transformed matrix *A*_**k**_ are stored in the manner indicated in panel (b.2).

For the sake of efficiency, these two schemes are combined. We here divide MPI processes into a two-dimensional grid schematically shown in Figure 2a, in which we show the assignment of *N*_p_ MPI processes to **k**-group and “row”. Each column corresponds to a **k**-point group consisting of *N*_r_ rows/MPI processes. **k**-dependent matrices, such as *H*_**k**_^KS^, are distributed within the MPI processes in the same column, where the assignment of blocks to *N*_r_ MPI processes is set the same for all columns in accordance with that of real-space matrices. The case of *N*_p_ = 4 MPI processes, *N*_g_ = 2, *N*_r_ = 2 is illustrated in Figure 2b. The *N*_r_ × *N*_g_ grid in this case is shown in Figure 2b.3. We consider a typical case of a real-space symmetric matrix *A* stored in the upper-triangular form. Provided that the blocks of *A* are assigned to 4 MPI processes as shown in Figure 2b.1, the blocks of its Fourier transformation *A*_**k**_ with **k** belonging to group 1 and group 2 are distributed as Figure 2b.2. With such block distribution, one can minimize MPI communications, which deteriorate the performance, in the following sense; (i) **k**-dependent matrix multiplications only require intra-**k**-group communications among *N*_r_ MPI processes in each column, whereas (ii) **k**-space integrations of matrices only require intrarow communications among *N*_g_ MPI processes in each row.

**Figure 3:**
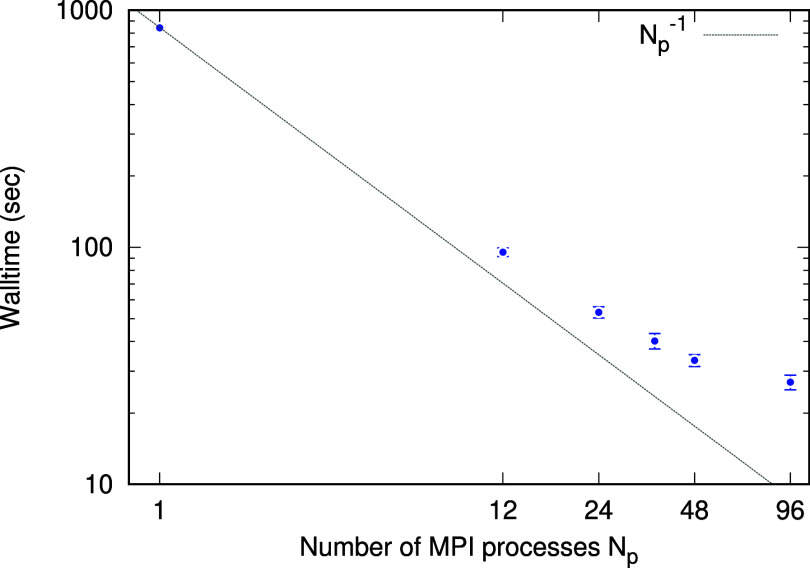
Step calculation times in parallel computation. Points show averaged step propagation time in the calculations with 1, 12, 24, 36, 48, and 96 MPI processes. Error bars show the statistical deviations from the averaged step propagation time. The thin dotted line shows the ideal scaling behavior proportional to *N*_p_^–1^ for reference.

We estimated the performance of our parallel computation scheme with benchmark calculations. Figure 3 shows the averaged single-step propagation time in optical absorption calculations of silicon. The system and the simulation procedure were the same as described in Subsection 2.2. We used the DZVP-GTH-PADE basis set^50^ and SIC-LDA *xc* functional of the Perdew–Zunger formulation.^51^ The Broullin zone was sampled using Monkhorst–Pack scheme^52^ with 16 × 16 × 16 **k**-point mesh reduced by the symmetry. The step size was set Δ*t* = 4 as. Parallel calculations in this subsection (Subsection 2.3) were performed on Piz-daint cluster^53^ at the Swiss National Supercomputing Centre. We calculated the same system with 1, 12, 24, 36, 48, and 96 MPI processes. The cluster machine we used in this computation has 12 cores and 12 MPI processes per cluster node. Each MPI process uses only 1 core. Calculations with 12, 24, 36, 48, and 96 MPI processes were performed using 1, 2, 3, 4, and 8 cluster nodes, whereas that with 1 MPI process was calculated using 1 node restricting the number of MPI processes and core to 1. Thus, the number of MPI processes in Figure 3 is also proportional to the size of computational resources in each calculation. In all but the single-MPI calculation, we propagated the TDKS orbitals for *N*_step_ = 51 steps (simulation time *T* = 204 as) whereas in the single-MPI calculation, we propagated for *N*_step_ = 7 steps (*T* = 28 as). We calculated the average of the step propagation times of the second to *N*_step_th steps excluding extraordinary data that deviates over 10 times the sample deviation (we found 2 such extraordinary data points in the whole data set). Error bars in Figure 3 indicate sample deviations among step propagation times.

From Figure 3, we see, up to 48 MPI processes, the computation time scales favorably with the number of processes *N*_p_ though the scaling is poorer than *N*_p_^–1^. This decrease in the parallel efficiency is attributed to the increased number of internode and/or interprocess communications at **k**-point integrations and real-space matrix manipulations. However, further detailed analyses for overcoming this limitation are left for future studies.

### 2.4 DFT + *U*

To extend our analysis to systems with strong electron correlations such as transition metal oxides, we need to go beyond LDA or the generalized gradient approximation (GGA), which are known to be insufficient for reproducing the ground-state electronic structures in those systems. It has been known that DFT + *U*(31,32) reproduces the ground-state electronic structures in those materials at a relatively low cost. DFT + *U* has also recently been applied to a wider range of systems to obtain correct band gaps^33^ or to fix the energy of filled bands relative to the Fermi level.^34^ In these studies, DFT + *U* is regarded as a method to fix the self-interaction problems^51,54^ existing in some semilocal *xc* functionals such as LDA and GGA with relatively low costs compared to the application of hybrid functionals. In excited-state calculation using RT-TDDFT, reproduction of the correct ground state and the band gaps are both critically important while the computational cost of each time step has to be minimized to realize long-time simulation. We therefore implemented DFT + *U* as a highly important extension of our RT-TDDFT.

In this paper, we work on the empirical formulation of DFT + *U*, where the effective on-site interaction for each atomic species,  in ref (32), is given as an input parameter. We extended the existing static DFT + *U* implementation in CP2K to static and real-time calculations with **k**-point sampling. Below, we first discuss several possible implementations of localized orbitals in the Gaussian-based calculations and derive expressions of projected density matrices, DFT + *U* energy corrections, and potential terms.

#### 2.4.1 Formulation of Static Calculations

We follow the formulation of Dudarev et al.^32^ We here label a set of orbitals by a symbol  and atomic index by *A* (in the case of ZnO calculations we discuss later,  represents “Zn 3d” or “O 2p” and the index *A* distinguishes two Zn or O atoms in the unit cell). The electronic spin index is represented by σ. The energy correction per unit cell is given as^32^
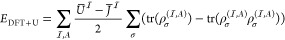
16where  and  formally represent the spherically averaged intra-atomic screened Coulomb repulsion and the Hund’s-rule exchange parameter,^31,32^ respectively, among the orbitals characterized by symbol . In practice,  works as an effective on-site repulsion term. The additional potential term is formally given as^32^
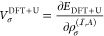
17with  representing the spin-dependent density matrix projected to the orbital set specified by .

The projection procedure is not unique and there are multiple methods proposed in the literature. The formulation/implementation of this part also depends on the basis sets used in the DFT program. Here, we consider two possibilities that can be relatively easily implemented in our Gaussian-based RT-TDDFT. We follow the idea developed by O’Regan et al.^37,38^ The projected density matrix is formulated as
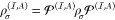
18where the spin-dependent density matrix in the coordinate representation is formally given as
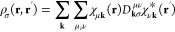
19with *D*_**k**σ_^μν^ being the spin-dependent density matrix in the AO representation. The projection operator  projects the density matrix onto a subspace spanned by a set of localized orbitals . The projection operator is written in the coordinate space as
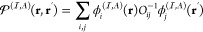
20where *O*_*ij*_^–1^ is the inverse of the overlap matrix among the orbitals in set .^37^ With this formulation, the projection operator is idempotent . Substitution of eqs 20 into 18 yields
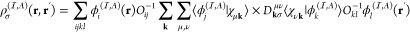
21

By defining a local projected density matrix  by
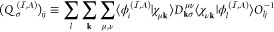
22we can calculate the trace of  (eq 21) as
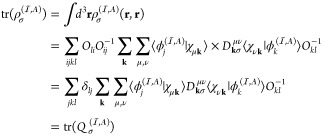
23awhere the last line follows from the definition of matrix  given in eq 22.

The other trace in eq 16 is also calculated in an analogous manner as
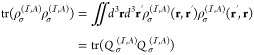
23b

The potential term, in the **k**-dependent AO basis representation, is calculated as
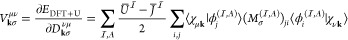
24with matrix  defined as
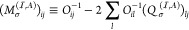
25

We note that our  corresponds to  in ref (38).

We then consider possible choices of local orbitals.

(1) Orthonormalized atomic orbitals (Löwdin orbitals)

Localized Löwdin orbitals^36^ are constructed from **k**-adapted AOs as
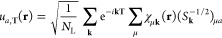
26with **T** being a lattice translation vector representing the cell coordinate of the orbital *u*_*a*,**T**_(**r**). These orbitals satisfy *u*_*a*,**T**_(**r** – **T**′) = *u*_*a*,**T**+**T**′_(**r**) and ⟨*u*_*a***T**_|*u*_*a*′**T**′_⟩ = δ_*aa*′_δ_**TT**′_. We can then define a set of orthogonalized orbitals as
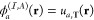
27where the lattice vector **T** is to be taken as the cell coordinate of the *A*th atom. With this orbital set, the overlap matrix is a unit matrix, and the overlap with χ_μ**k**_(**r**) is given as
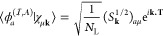
28and  is given as (we use indices *a*, *b*, ... instead of *i*, *j*, ... as in eq 22)
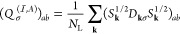
29

As it should be, the RHS of eq 29 is independent of the cell coordinate **T**. We note that the localized Löwdin orbitals defined as eq 26 are not necessarily localized in a single unit cell. By substituting the definition of **k**-adapted Gaussian orbital given in eqs 3 into 26, we obtain another expression of *u*_*a*,**T**_(**r**)
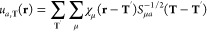
30with  being the inverse Fourier transformation of *S*_**k**_^–1/2^. Equation 30 indicates that *u*_*a*,**T**_(**r**), in general, extends over multiple cells.

(2) Orbitals of an isolated atom^38^

As formulated by Chai et al.,^38^ a reasonable choice of localized orbitals is the orbitals of an isolated atom of the target atomic species, represented as
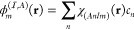
31where (**r**) are AOs belonging to the *A*th atom with angular and azimuthal quantum number  and *m*. The angular momentum  is fixed by the orbital character . The index *n* distinguishes AOs in the basis set with the same  and *m*, and the linear combination coefficient *c*_*n*_ is computed by a separate ground-state DFT calculation of an isolated atom using the same Gaussian basis set. The overlap matrix of such local orbitals becomes a unit matrix. The overlap with χ_μ**k**_(**r**) is given as
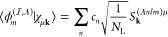
32and  is given as (we use indices *m*, *m*′ instead of *i*, *j* as in eq 22)
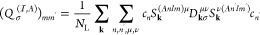
33

Scheme (2), which is an extension of the formulation by Chai et al.,^38^ has favorable properties that (i) the localized orbitals  are formally independent of the choice of the basis set in the sense they converge to the exact (within the approximation introduced by the *xc* functional) KS orbitals of an isolated atom in the limit of the complete basis set, (ii) the number of orbitals is fixed at 2 + 1 (i.e., the number of orbitals in an isolated atom with angular momentum ), independent of the size of the basis set, and (iii)  are, by construction, localized in space. On the other hand, a set of Löwdin orbitals lacks these properties. Examples of these local orbitals in schemes (1) and (2) are shown in the Supporting Information [Figures S1 and S2 shows those of scheme (1) and Figure S3 shows those of scheme (2)].

#### 2.4.2 Formulation of Dynamical Calculations

As was pointed out by Tancogne-Dejean et al.,^35^ the potential term in DFT + *U* has a nonlocal character. We therefore need to apply the gauge transformation in the simulations with the velocity-gauge formulation of external fields. In general, gauge transformation with a spatially uniform vector field **A**_*t*_, , accompany a transformation of the nonlocal operator  where *q*_e_ and *c* represent the electron charge and the speed of light, respectively,

The nonlocality of the DFT + *U* potential term also introduces a correction term for the velocity operator
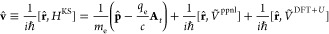
34where **p̂** is the momentum operator, *m*_e_ represents the electron mass, and *V*^ppnl^ represents the nonlocal part of the pseudopotential. To calculate the matrix elements of gauge-transformed  and the commutator , we first introduce an explicit expression of the coordinate representation of the nonlocal potential
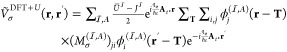
35

The matrix elements of commutator  is calculated as
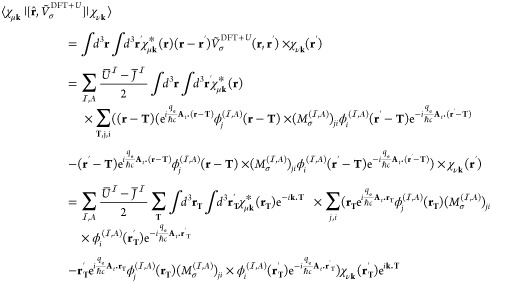
36where in the last two line, we changed the integration variables **r** and **r**′ to **r**_**T**_ ≡ **r** – **T** and **r**_**T**_^′^ ≡ **r**^′^ – **T**, respectively. We also used the property of **k**-adapted Gaussian AOs χ_μ**k**_(**r**) = χ_μ**k**_(**r**_**T**_ + **T**) = χ_μ**k**_(**r**_**T**_)e^*i***k**·**T**^. By introducing notations
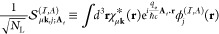
37and
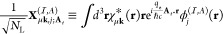
38which are both finite due to the finiteness of the support of , we can rewrite eq 36 as
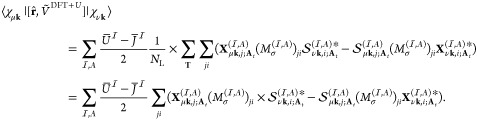
39

The matrix elements of  are also calculated in the same manner as
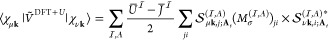
40

In numerical calculations, in scheme (1), the matrix  is calculated as
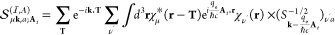
41whereas **X** is calculated as
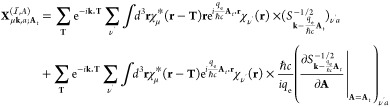
42where the derivative  is calculated using numerical differentiation. To get eq 42, we used the property
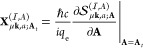
43which directly follows from eqs 37 and 38. In scheme (2), they are calculated as
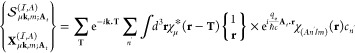
44

## 3 Calculation Results

### 3.1 Optical Absorption Spectrum of Solids

We here discuss the optical absorption spectrum of linearly polarized light. We adopt the velocity gauge formulation to incorporate an external field into our simulation. We use a step function vector potential **A**(*t*) = −*c***n***F*_0_θ(*t*) with *F*_0_ and **n** being the amplitude and polarization. θ(*t*) represents the step function. This vector potential corresponds to an impulsive electric field **E**(*t*) = **n***F*_0_δ(*t*) in the length gauge formulation. We evaluate the velocity operator  along the time evolution. In this paper, we set *F*_0_ = 0.001 au and **n** in the *z*-direction unless specified otherwise.

The dielectric function ε(ω) is then computed by the formula ε(ω) = 1 + 4π*i*σ(ω)/ω using the dynamical conductivity  with  being the Fourier transformation of the calculated velocity and Ω_c_ being the cell volume. To get the Fourier spectra of the observed quantities, we used an approximation
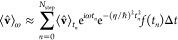
45where *t*_*n*_ is the *n*th time point,  is the expectation value of the operator **v̂** per unit cell at time point *t*_*n*_, η is a line-broadening factor, *N*_step_ is the number of time steps, and *f*(*t*) is a damping function. In the calculation shown below, We used η = 0.1 eV and the third-order polynomial damping function^55^*f*(*t*) = 1 + 2(*t*/*T*)^3^ – 3(*t*/*T*)^2^ with *T* = Δ*tN*_step_ being the total simulation time.

**Figure 4:**
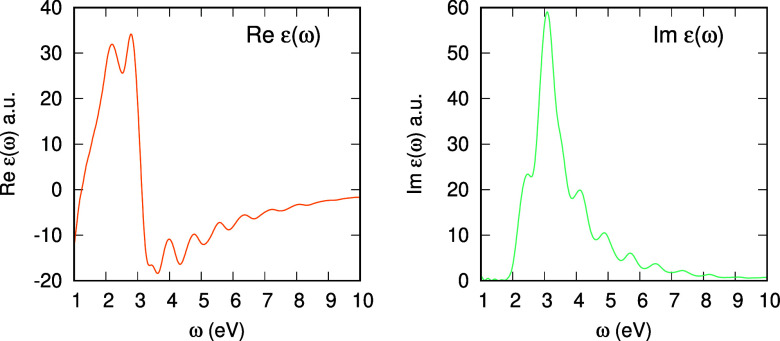
Real and imaginary parts of the dielectric function ε(ω) of bulk silicon.

As a benchmark application, we calculated the dielectric function of bulk silicon. The system and the simulation procedure were the same as described in Section 2.2. We used the DZVP-GTH-PADE basis set^50^ and SIC-LDA *xc* functional of the Perdew–Zunger formulation.^51^ The time step was taken Δ*t* = 4 as and the TDKS orbitals were propagated for 20 fs (5000 steps). The Brillouin zone was sampled using the Monkhorst–Pack^52^**k**-mesh of 17 × 17 × 17. Figure 4 shows the real and imaginary parts of the dielectric function ε(ω).

We find the peak of Imε(ω) at around ℏω ≈ 3.1 eV, which is smaller than ℏω ≈ 3.8 eV indicated in ref (56). Since our **k**-point sampling is Γ-centered and the density is slightly larger than that used in ref (56), Γ-centered 16 × 16 × 16, we can exclude **k**-sampling as a main cause of this difference. To examine the possibility of a wrong implementation of RT-TDDFT, we checked consistency of the results of our RT-TDDFT with those of the linear-response TDDFT within the Γ-point calculation with the same computational setting as reported in the Supporting Information.

We therefore attribute this difference in the peak positions to the differences in the basis sets and pseudopotentials, among others.

### 3.2 DFT + *U*

#### 3.2.1 Static Calculation Results

We extended the existing static DFT + *U* codes in CP2K, which was only for Γ-point calculations, to **k**-sampling calculations, with two local orbital projection schemes we discussed in Subsection 2.4. As a benchmark of our DFT + *U* implementation, we applied it to the calculation of wurtzite ZnO. The band gap of this material has been experimentally derived as Δ_g_ = 3.44 eV by optical absorption^57^ and reflectance,^58^ Δ_g_ = 3.3 eV by X-ray absorption spectroscopy.^59^ Theoretical results include Δ_g_ = 2.11 eV^60^ by calculations using the range-separated hybrid functional formulated by Heyd, Scuseria, and Ernzerhof (HSE03)^61^ and Δ_g_ = 3.33 eV by HSE03-based GW calculations.^60^ The DFT + *U* studies of ZnO include a detailed study by.

Ma et al. in ref (62), in which they applied Hubbard *U* to oxygen 2p orbitals as well as zinc 3d orbitals to reproduce the band gap close to the experimental value 3.4 eV. We here calculated wurtzite ZnO with lattice constant *a* = 3.125 Å using PBE^63^ + *U* with the effective on-site potentials *U*^eff^ (corresponding to  in Subsection 2.4) set to *U*_Zn 3d_^eff^ = 10.0 eV and *U*_O 2p_^eff^ = 7.0 eV, for zinc 3d orbitals and oxygen 2p orbitals, respectively. These values were chosen so that, according to Figure 4 in ref (62), their projected augmented wave (PAW) calculation should reproduce the band gap 3.4 eV. We applied the *U*-ramping^64^ technique to get convergent results. We used 3-step ramping where the values of (*U*_Zn 3d_^eff^, *U*_O 2p_^eff^) were increased as (3.3334, 2.3334), (6.6668, 4.6668), (10.000, 7.000) eV. At each intermediate ramping step, the SCF iterations were repeated until we reached an intermediate convergence threshold ϵ_R_ = 1.0 × 10^–4^. We confirmed the convergence of the values of the band gap with respect to the number of ramping steps by comparing the results with those of 8-step ramping calculations. We used GTH pseudopotential^49^ for PBE (GTH-PBE) implemented in CP2K. The **k**-points were sampled with Monkhorst–Pack 15 × 15 × 15 mesh reduced by the crystal symmetry. In Table 1, we show the results of static calculations. We used MOLOPT basis sets^65^ with varying sizes; (i) DZVP-MOLOPT-SR-GTH with 76 AOs (Zn:2s2p2d1f and O:2s2p1d) per unit cell, (ii) TZVP-MOLOPT-PBE-GTH with 102 AOs (Zn:3s3p3d1f and O:3s3p1d), and (iii) TZV2P-MOLOPT-PBE-GTH with 140 AOs (Zn:3s3p3d2f and O:3s3p2d1f). With scheme (1) (see Subsection 2.4), the obtained values of the band gap were much smaller than the experimental value and they substantially decreased with the size of the basis set. We attribute this problem in scheme (1) to the increase in the projection space with the size of the basis set (the projected space spanned by Zn d orbitals, for example, becomes larger as the number of AOs of Zn d character in the basis set increases). The scheme (2), on the other hand, was found to be more robust against the size of the basis set and the obtained value is closer to the experimental (optical absorption/reflection) value 3.44 eV or the result obtained by Ma et al.^62^ 3.4 eV. The difference from the latter DFT + *U* result obtained using the same *xc* functional is attributed to the differences in the projection scheme, basis sets, pseudopotentials, etc. We here consider that the qualitatively good agreement with those previously obtained results supports the validity of scheme (2).

**Table 1:** Band Gaps of Wurtzite ZnO Calculated Using Two Projection Schemes Introduced in Section 2.4^a^

	DZVP-MOLOPT-SR	TZVP-MOLOPT	TZV2P-MOLOPT
scheme (1)	2.075	0.701	0.640
scheme (2)	3.227	3.173	3.172

a Values are shown in the eV unit.

**Figure 5:**
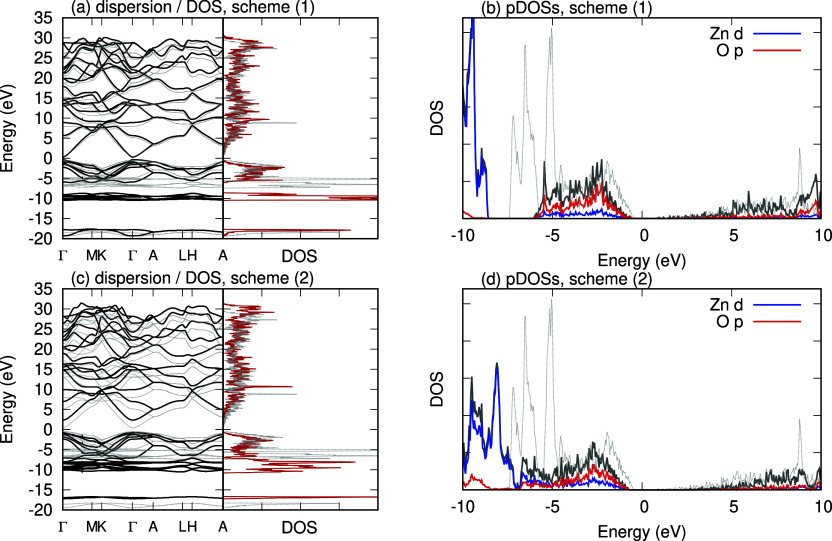
Static calculation results of the band dispersion and DOS of wurtzite ZnO. Panels (a,c) show the band dispersion and total DOS whereas panels (b,d) show the partial DOSs (pDOSs) projected to Zn d (blue lines) and O p (red lines) orbitals as well as total (gray solid lines) DOS in the energy range −10 to 10 eV measured from the Fermi level. In each panel, the thin gray dotted line shows the dispersion [panels (a,c)] or the total DOS [panels (a–d)] calculated using PBE (without *U*). Panels (a,b) show the results obtained using scheme (1) and panels (c,d) show those obtained using scheme (2).

The robustness of scheme (2) is understood from the fact that in scheme (2), the projected space is always expanded with a fixed number of orbitals and these orbitals have fixed limiting forms with respect to the size of the basis set (as discussed in Subsection 2.4, they converge to the exact KS orbitals for a given *xc* functional in the limit of complete basis set). The results calculated using the TZV2P-MOLOPT-PBE-GTH basis set are shown in Figure 5. We show the band dispersion and the density of states in panels (a,c). The partial DOSs of zinc d orbitals and oxygen p orbitals calculated from the Mulliken population analysis of KS orbitals are shown in panels (b,d). Panels (a,b) are the results of the calculation using projection scheme (1) whereas panels (c,d) are those of the calculation using projection scheme (2). In each figure, we also plotted the corresponding quantities obtained using PBE (without *U*) for comparison.

As shown in Figure 5a,b, we find a vanishing gap in the results obtained using scheme (1) while in Figure 5c,d we find a clear band gap in the results obtained using scheme (2). Comparison of dispersions in Figure 5a,c clarifies the difference between those schemes. We find that, while the conduction band dispersion near the Fermi level (ε = 0) obtained using scheme (1) is close to that of PBE without *U*, that obtained using scheme (2) is shifted upward, resulting in a finite band gap. It is known that,^32,66^ and is implicitly indicated in eq 25, if the localized orbitals are a good representation of relevant bands, the Hubbard *U* term lowers (raises) occupied (unoccupied) bands by . The behavior seen in the result of scheme (2) is therefore close to what was expected in DFT + *U* calculations.

#### 3.2.2 Dynamical Calculation Results

We next calculated a linear optical absorption spectrum of the same material (wurtzite ZnO *a* = 3.125 Å) using our RT-TDDFT. We used PBE + *U* with *U*_Zn 3d_^eff^ = 10.0 eV and *U*_O 2p_^eff^ = 7.0 eV. We used GTH-PBE pseudopotential and DZVP-MOLOPT-SR-GTH basis set. The optical response is calculated in the velocity gauge formulation as described in Section 3.1, the step size was set Δ*t* = 8 as and we propagated the TDKS orbitals for 20 fs (2500 steps). In this calculation, the Brillouin zone was sampled using the Monkhorst–Pack^52^**k**-mesh of 6 × 6 × 6. At the initial ground state calculation at *t* = 0, we applied a 4-step *U*-ramping scheme where the values of (*U*_Zn 3d_^eff^, *U*_O 2p_^eff^) were increased as (2.50, 1.75), (5.00, 3.50), (7.50, 5.25), (10.00, 7.00) eV. The convergence of this ramping scheme was checked by comparison to the 8-step ramping calculation results.

**Figure 6:**
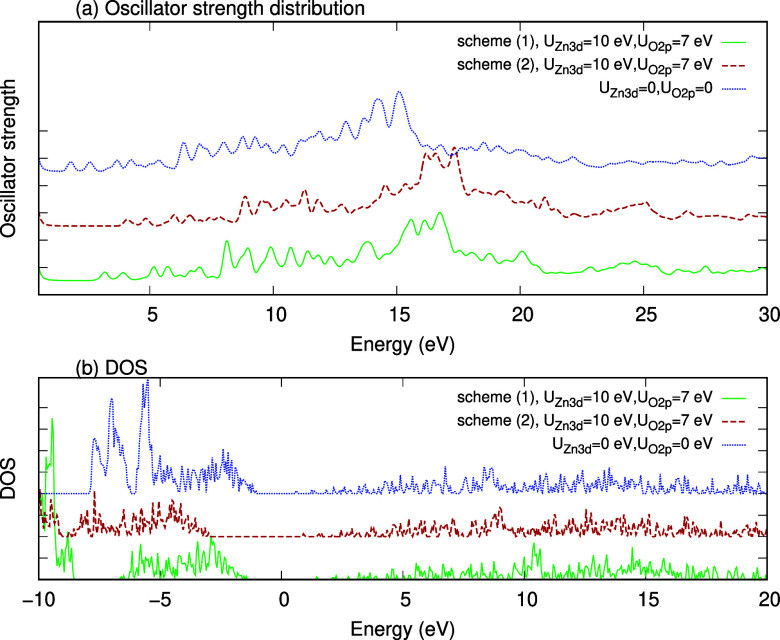
Optical absorption spectrum and static properties of wurtzite ZnO. Lines are shifted vertically for clarity. Panel (a) shows the oscillator strength distributions calculated using PBE + *U* with schemes (1) and (2) with green solid and red dashed lines, respectively. We also plot, the results obtained without *U* with blue dotted lines. Panel (b) shows the local DOS obtained by a static calculation using each scheme.

The results were analyzed in the same way shown in Section 3.1. Here we use the oscillator strength distribution calculated as
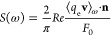
46for analysis. Here we discuss consistency between two types of gaps. One is the band gap Δ_g_, which is obtained from a static ground-state calculation as the difference of the lowest KS orbital energy above the Fermi energy and the highest KS orbital energy below the Fermi energy. The other one is the optical gap Δ_o_, which is estimated by the energy of the lowest energy peak in the oscillator strength distribution. The values of the optical gap Δ_o_, estimated from the oscillator strength distributions shown in Figure 6a are Δ_o_ ≈ 3.2 eV for the scheme (1) and Δ_o_ ≈ 4.1 eV for the scheme (2), while the band gaps in the static calculations were Δ_g_ = 2.933 eV for scheme (1) and Δ_g_ = 3.930 eV for scheme (2).^67^ In principle, the optical gap Δ_o_, estimated from the lowest energy optical transition should be smaller than the band gap by the exciton binding energy. We, however, do not consider this as a severe problem since (i) our Δ_o_ and Δ_g_ are obtained from different levels of theory, (RT-)TDDFT + *U* and ground-state DFT + *U*, (ii) the exciton binding energy is known to be ≈ 0.06 eV^57,58^ and we cannot expect our simulation using the PBE *xc* potential, which lacks the correct exchange tail, to be able to reproduce such small exciton energy. We here focus on the point that, in both schemes, we obtained reasonable agreement between Δ_g_ obtained from static DFT + *U* calculations and Δ_o_ from RT-TDDFT + *U* calculations. This consistency supports the validity of our RT-TDDFT + *U* implementation. More detailed analyses of the difference Δ_o_ and Δ_g_, which require the reproduction of exciton binding energies, are left for future studies using more accurate *xc* functionals.

## 4 Summary

We developed RT-TDDFT with **k**-point sampling in CP2K. In addition to standard TDKS orbital propagation codes, we implemented **k**-point reduction and **k**-point parallelization, which are critically important for efficient calculations and we regarded the implementation of them as a necessary task for Gaussian-based RT-TDDFT to be of practical use. As shown in the results of benchmark calculations, we successfully implemented **k**-point reduction in Gaussian-based RT-TDDFT and we also achieved reasonable scaling of the step-propagation time with the number of MPI processes. We also have implemented DFT + *U* as a useful extension. We found that the formulation of the projector proposed by Chai et al.^38^ worked favorably in **k**-point sampling codes. The superiority of their projection scheme (scheme (2) in Section 2.4) over the Löwdin orbital projection scheme (scheme (1) in Section 2.4) was attributed to the fact that the projected space in scheme (2) is expanded with a fixed number of orbitals that have fixed limiting forms with respect to the size of the basis set (see discussions in Subsections 2.4 and 3.2.1). Such robustness and smallness of additional computational costs are both favorable for real-time calculations. To our knowledge, our scheme (2) is the first extension of the “tensorial” subspace representation^37,38^ approach in DFT + *U* to **k**-point sampling RT-TDDFT, and the result indicates that it is a promising approach to RT-TDDFT + *U* calculations. This result also indicates the usefulness of the localized nature of the Gaussian basis set, which applies to isolated systems as well as periodic systems. In the GPW framework, one can make use of this locality of the Gaussian basis set while realizing efficient computation of the Hartree potential in periodic systems by utilizing the auxiliary plane wave basis set.^23,24^

Having developed the basic part of **k**-point sampling RT-TDDFT which is feasible for large-scale calculations, our next goal is to extend it to real-time dynamics possibly coupled with nuclear dynamics in general nonperturbative dynamics. The localized nature of the Gaussian basis set might especially work favorably in nonbulk systems such as surfaces with adsorbed molecules.

## Figures and Tables

**Figure 7:**
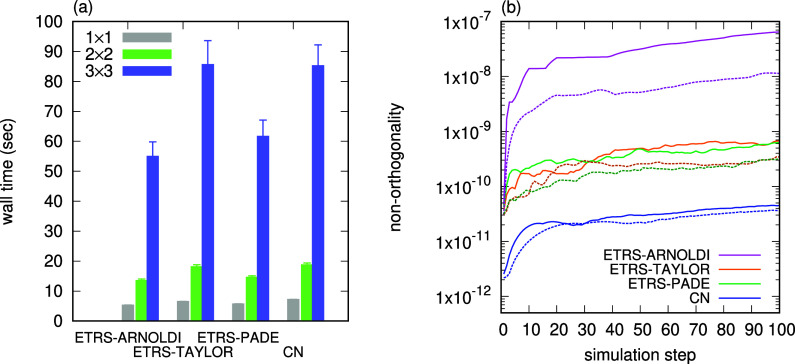
Comparison among 4 propagation schemes, (a) time performance and (b) accuracy. In panel (a), we plotted the average step-propagation times with 4 propagation schemes in gray, green, and blue boxes for *n* = 1, 2, 3 supercells. Error bars show sample standard deviations. In panel (b), we plotted the numerical nonorthogonality as a function of the propagation step. Solid lines show the errors in simulations with step size Δ*t* = 8 whereas dashed lines show those with step size Δ*t* = 4 as. Pink, orange, green, and blue lines correspond to ETRS-ARNOLDI, ETRS-TAYLOR, ETRS-PADE, and CN, respectively.

## Data Availability

The data that support the findings of this study are available from the corresponding author upon reasonable request.

## Appendix A

### Comparison among Propagators

We implemented the CN propagator and the ETRS propagator with 3 exponential computation schemes (the Arnoldi iteration algorithm, Padé approximation, and Taylor expansion). We tested the time performance and accuracy of these 4 propagation schemes. To test the time performance, we performed velocity-gauge optical absorption spectrum calculations with *n* × *n* supercells of h-BN with *n* = 1, 2, 3. Here, we set the polarization of the external field **n** in the *x*-direction. Other details of optical absorption spectrum calculations are the same as we described in Section 3.1.

We used the DZVP-GTH basis set given in PySCF^68^ software and the PADE LDA functional^49^ implemented in CP2K. We used GTH-PADE pseudopotential and **k**-points were sampled from the irreducible Brillouin zone of Monkhorst–Pack^52^**k**-meshes of 9 × 9 × 1. Step size was set Δ*t* = 4 as. We propagated the TDKS orbitals for *N*_step_ = 100 steps (simulation time *T* = 400 as).

The averaged walltimes for a single-step propagation are shown in Figure 7a, which show no significant difference for small cells as *n* = 1, 2. We next tested the accuracy of the propagators by checking the numerical nonorthogonality of TDKS orbitals. In Figure 7b, we plotted the numerical nonorthogonality (the maximum values of |⟨ψ_**k**λ_|ψ_**k**λ′_ – δ_λ,λ′_|) taken from *n* = 1 unit cell calculation described above (dashed lines), and the same calculations with larger step size Δ*t* = 8 as (solid lines). The results show that the CN is the most accurate among the 4 schemes.

## Appendix B

### Derivation of the AO Rotation Formula eq 11

Here we show the derivation of the AO rotation formula eq 11. Below, as in the main text, the atomic center of AO χ_μ_ in the unit cell at the origin is denoted as **R**_*A*_μ__, whereas its angular momentum and the azimuthal quantum number around the atomic center are denoted as _μ_ and *m*_μ_, respectively. The atomic species of atom *A*_μ_ is denoted as [*A*_μ_]. Since AO χ_μ_(**r**) is uniquely specified by its atomic center coordinate **R**_*A*μ_, atomic species [*A*_μ_], angular quantum numbers (_μ_, *m*_μ_) and an additional index *n*_μ_, we can rewrite it as

47

where  represents the *n*_μ_th AO of atomic species [*A*_μ_] with angular quantum numbers (_μ_, *m*_μ_) as a function of the coordinate relative to the atomic center **x**. Using these notations, the rotation of AO χ_μ**k**_(**r**) is calculated as

48

where in the fifth line, we used the index transformation eq 9c, whereas in the sixth line, we used the fact  and introduced . In the sixth line, we switched our notation from ξ to χ; an AO centered at atom *f*_{α|**b**}_(*A*_μ_) specified by the indices (*n*_μ_, _μ_, *m*′) is rewritten as χ_μ′_(**r**). We note that the atomic species of atom *f*_{α|**b**}_(*A*_μ_) equals [*A*_μ_] by symmetry. Summation over μ′ in the seventh and eighth lines are to be taken over 2 _μ_ + 1 atomic orbitals centered at *f*_{α|**b**}_(*A*_μ_), index *n*_μ_, and the angular momentum _μ_. In the main text, for notational simplicity,  is denoted as .

## Supporting Information Available

Local orbitals in Scheme (1) (Figures S1 and S2); Local orbitals in Scheme (2) (Figure S3); Comparison between LR-TDDFT and RT-TDDFT results (Figure S4) (PDF)
