# Supplementary material for: Development of Real-Time TDDFT Program with k-Point Sampling and DFT + U in a Gaussian and Plane Waves Framework
Source: J Chem Theory Comput. 2025 Feb 8;21(4):1879–91. doi: 10.1021/acs.jctc.4c01515 (PMC11866758; doi:10.1021/acs.jctc.4c01515)
Supplement: Supplementary file 1 — ct4c01515_si_001.pdf [file ct4c01515_si_001.pdf]

SUPPORTING INFORMATION FOR Development of real-time TDDFT program with k-point sampling and DFT+U in a Gaussian and plane waves framework

Kota Hanasaki* and Sandra Luber

Department of Chemistry, University of Zurich, 8057 Zurich, Switzerland

E-mail: kota.hanasaki@chem.uzh.ch

S1 Local orbitals in scheme (1)

We show examples of local orbitals in scheme (1) we discussed in Section 2.4. Our main focus is in their spatial distribution over (multiple) unit cells. In scheme (1), local orbitals are Löwdin orbitals defined as (eq (26) in the main text)

$$u_{a,\mathbf{T}}(\mathbf{r}) = \sqrt{\frac{1}{N_L}} \sum_{\mathbf{k}} e^{-i\mathbf{k}\mathbf{T}} \sum_{\mu} \chi_{\mu\mathbf{k}}(\mathbf{r}) \left(S_{\mathbf{k}}^{-1/2}\right)_{\mu a}, \tag{S1}$$

which is rewritten in a real-space summation (eq (30) in the main text)

$$u_{a,\mathbf{T}}(\mathbf{r}) = \sum_{\mathbf{T}'} \sum_{\mu} \chi_{\mu}(\mathbf{r}-\mathbf{T}') S_{\mu a}^{-1/2}(\mathbf{T}-\mathbf{T}'). \tag{S2}$$

To show the approximate shapes of these Löwdin orbitals, we calculated eq (S2) with $\mathbf{T} = \mathbf{0}$ in $N_1 \times N_2 \times N_3$ supercell with N_1, N_2, and N_3 being odd integers, i.e. we restricted the

summation over $\mathbf{T}'$ in eq (S2) to a subset of lattice vectors $\mathcal{T}_{N_1\times N_2\times N_3} \equiv \{\sum_{j=1}^{3} h_j \mathbf{a}_j | |h_1| \leq (N_1-1)/2, |h_2| \leq (N_2-1)/2, |h_3| \leq (N_3-1)/2\}$ with $\mathbf{a}_j$ being the jth primitive translation vector and h_j being its associated integer coefficient. Since these approximate orbitals are not normalized because of the truncation, we explicitly normalized them as

$$u_{a,\mathbf{0}}(\mathbf{r}) \approx \frac{\sum\limits_{\mathbf{T}'\in\mathcal{T}_{N_1\times N_2\times N_3}} \sum\limits_{\mu} \chi_\mu(\mathbf{r}-\mathbf{T}') S_{\mu a}^{-1/2}(-\mathbf{T}')}{\sqrt{\int d^3\mathbf{r}' \left| \sum\limits_{\mathbf{T}'\in\mathcal{T}_{N_1\times N_2\times N_3}} \sum\limits_{\mu} \chi_\mu(\mathbf{r}'-\mathbf{T}') S_{\mu a}^{-1/2}(-\mathbf{T}') \right|^2}}$$
$$= \frac{\sum\limits_{\mathbf{T}'\in\mathcal{T}_{N_1\times N_2\times N_3}} \sum\limits_{\mu} \chi_\mu(\mathbf{r}-\mathbf{T}') S_{\mu a}^{-1/2}(-\mathbf{T}')}{\sqrt{\sum\limits_{\mathbf{T}'_1,\mathbf{T}'_2\in\mathcal{T}_{N_1\times N_2\times N_3}} \sum\limits_{\mu\nu} S_{a\mu}^{-1/2}(\mathbf{T}'_1) S_{\mu\nu}(\mathbf{T}'_2-\mathbf{T}'_1) S_{\nu a}^{-1/2}(-\mathbf{T}'_2)}}. \tag{S3}$$

To plot orbitals, we computed amplitudes of each orbital $u_{a,\mathbf{0}}(\mathbf{r})$ on a uniform spatial grid with spacing Δr_{grid} and derived the threshold amplitude $A_{0.5}$ such that $\Delta V_{\text{grid}} \sum\limits_{\substack{\mathbf{r}_j\in\text{grid} \\ |u_{a,\mathbf{0}}(\mathbf{r}_j)|^2 \geq A_{0.5}^2}} |u_{a,\mathbf{0}}(\mathbf{r}_j)|^2 = 0.5\Delta V_{\text{grid}} \sum\limits_{\mathbf{r}_j\in\text{grid}} |u_{a,\mathbf{0}}(\mathbf{r}_j)|^2$, with $\Delta V_{\text{grid}} = (\Delta r)^3$ being the volume element of the numerical grid. We then plotted the approximate equiamplitude surface at $u_{a,\mathbf{0}}(\mathbf{r}) = \pm A_{0.5}$ using interpolation of amplitudes on grid points $\{u_{a\mathbf{0}}(\mathbf{r}_j)\}$. By such construction, the inner region $\mathcal{V}_{0.5}$ enclosed in those surfaces contains approximately *the half of the target orbital*, in the sense $\int_{\mathcal{V}_{0.5}} |u_{a,\mathbf{0}}(\mathbf{r})|^2 d^3\mathbf{r} \approx 0.5$. Graphics of equiamplitude surfaces were generated using the Jmol software.[1]

Here we took an example in the calculation of ZnO we discussed in Section 3.2 in the main text. We calculated localized orbitals of wurtzite ZnO with lattice constant $a = 3.125$ Å using TZV2P-MOLOPT-PBE-GTH basis set,[2] sampling the Brillouin zone with Monkhorst-Pack[3] $15 \times 15 \times 15$ mesh. We used (i) supercells $\mathcal{T}_{3\times3\times3}$ and grid spacing $\Delta r_{\text{grid}} = 0.15$ a.u. for plotting local orbitals with angular momentum $\ell = 2$ centered at a zinc atom and (ii) $\mathcal{T}_{5\times5\times5}$ and grid spacing $\Delta r_{\text{grid}} = 0.30$ a.u. for orbitals with $\ell = 1$ centered at an oxygen atom.

In Figures S1 and S2, we plotted (i) 10 local orbitals centered at a zinc atom with

angular momentum $\ell = 2$ and (ii) 6 local orbitals centered at an oxygen atom with angular momentum $\ell = 1$, respectively. We note that some of the orbitals extending over multiple unit cells lack symmetry expected from the lattice symmetry because of the truncation of summation and subsequent normalization shown in eq (S3). The purpose of these plots is to illustre the extended characters of these orbitals.

From Figures S1 and S2, we find that, while one set of $2\ell + 1$ orbitals are almost localized in a single unit cell, other sets of orbitals extend over multiple unit cells. We can understand such an extended character arises from mutual orthogonality of Löwdin orbitals (see discussion in main text subsection 2.4.1). We can then anticipate that multiple sets of atomic orbitals with the same character (atomic center and the angular momentum) in multi-zeta basis set yields such extended Löwdin orbitals. This should be closely related to the vanishing gap in the calculations using scheme (1) and large basis sets (see Table 1 in the main text).

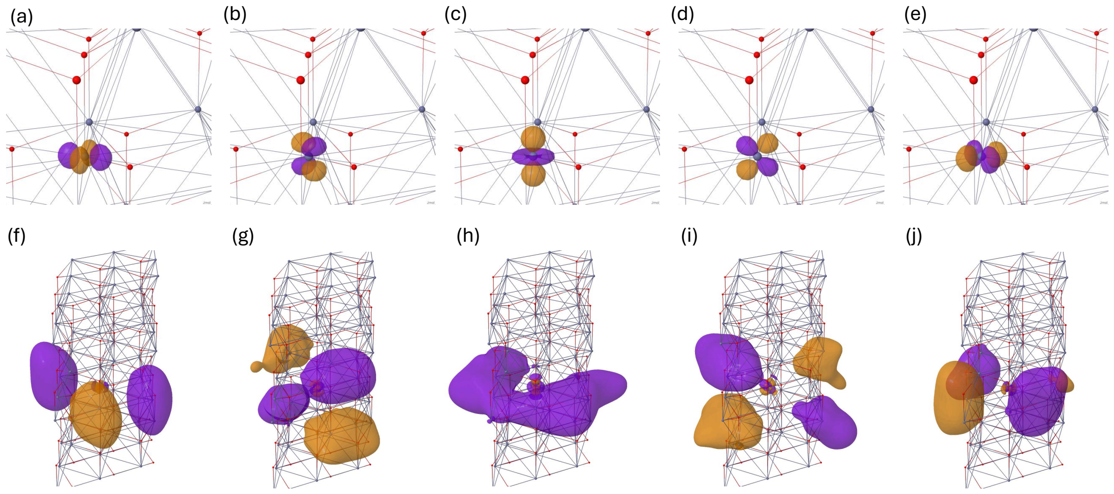

Figure S1: Local orbitals centered at a zinc atom with angular momentum $\ell = 2$ in scheme (1). The blue-gray and red spheres represent Zn and O atoms, respectively. Orange and purple surfaces represent the equiamplitude surfaces of orbitals at $A_{0.5}$ and $-A_{0.5}$, respectively.

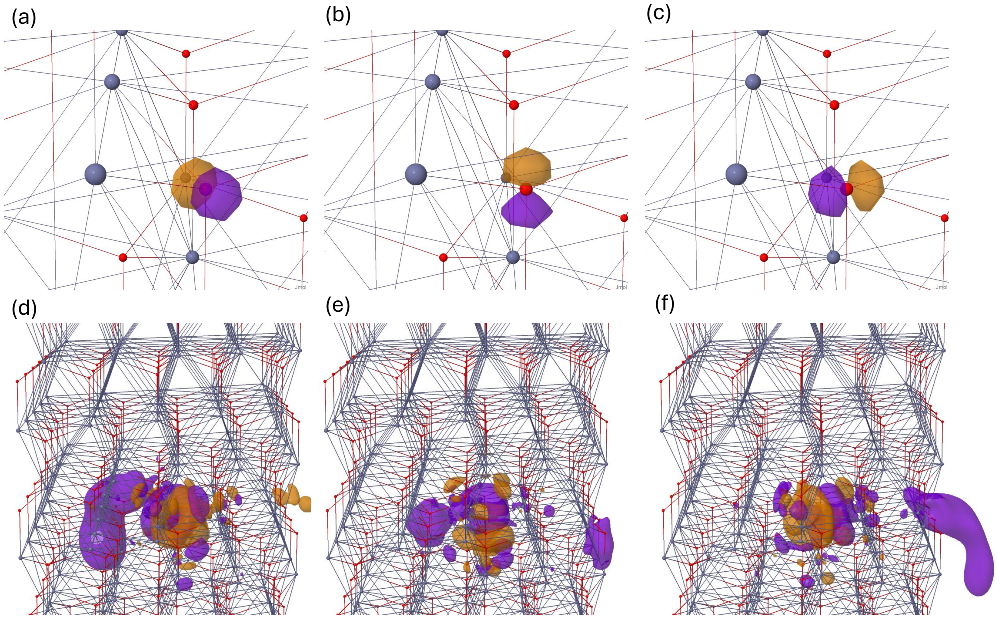

Figure S2: Local orbitals centered at an oxygen atom with angular momentum $\ell = 1$ in scheme (1). Conventions are the same as those in Fig. S1.

S2 Local orbitals in scheme (2)

To illustrate the difference, we also show the local orbitals in scheme (2). The local orbitals in scheme (2) are defined as (eq (31) in the main text)

$$\phi_m^{(\mathcal{I},A)}(\mathbf{r}) = \sum_n \chi_{(An\ell m)}(\mathbf{r}) c_n, \tag{S4}$$

and they are independent of **k**-point sampling. We plotted these local orbitals for TZV2P-MOLOPT-PBE-GTH basis set.[2] The equiamplitude surfaces $\phi_m^{(\mathcal{I},A)}(\mathbf{r}) = \pm A_{0.5}$ are calculated in the same manner as Section S1. Local orbitals in this scheme can be calculated using a single unit cell, however, in Figure S3, we plotted atoms in neighboring unit cells as well for better comparison to Figures S1 and S2.

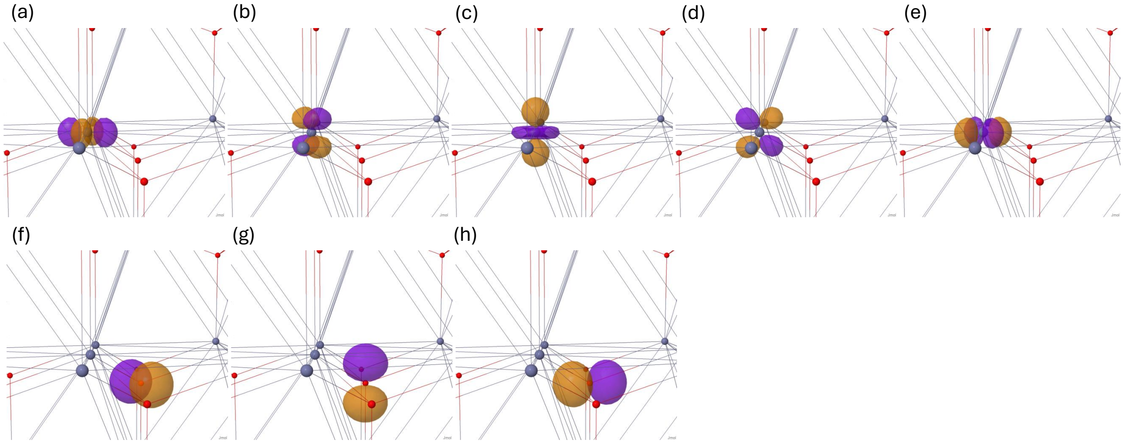

Figure S3: Local orbitals in scheme (2). Panels (a)-(e) show the local orbitals centered at a zinc atom with angular momentum $\ell = 2$, whereas panels (f)-(h) show the local orbitals centered at an oxygen atom with angular momentum $\ell = 1$. Other conventions are the same as figure S1.

S3 Comparison between LR-TDDFT and RT-TDDFT results

In order to validate our implementation of RT-TDDFT, we compared the results of RT-TDDFT calculations and those of linear-response TDDFT (LR-TDDFT) calculations with the same computational settings. In CP2K, $\mathbf{k}$-point sampling LR-TDDFT is not yet implemented while the Γ-point LR-TDDFT in the Tamm-Dancof approximation[4] is available in its Time-Dependent Density Functional Perturbation Theory (TDDFPT) program.[5] We therefore compared the Γ-point LR-TDDFT calculation and our $\mathbf{k}$-point sampling RT-TDDFT using the Monkhorst-Pack[3] $\mathbf{k}$-mesh of $1 \times 1 \times 1$. Following Section 3.1, we calculated the optical absorption spectrum of silicon. The primitive unit cell of silicon is taken as an 8-atom cubic cell with the lattice constant 5.4306975 Å. We then constructed (a) $2 \times 2 \times 2$ and (b) $2 \times 2 \times 4$ supercells consisting of 64 and 128 Si atoms. Other calculation conditions are the same as those in section 3.1; we used SIC LDA formulated Perdew and Zunger[6] and Goedecker-Teter-Hutter (GTH) pseudopotential,[7] we used DZVP-GTH-PADE basis set. In RT-TDDFT calculations, we propagated TDKS orbitals for (a) $N_{\mathrm{step}} = 5000$ steps with

step size $\Delta t = 4$ fs, and (b) $N_{\mathrm{step}} = 2500$ steps with step size $\Delta t = 8$ fs. In LR-TDDFT calculations, we calculated (a) 2400 states, and (b) 12000 states, respectively. We calculated the oscillator strength distribution $S(\omega)$ from RT-TDDFT calculation results as we described in the main text subsection 3.2.2., whereas that of LR-TDDFT is calculated as $S_j = \frac{2}{3}\Delta E_j \sum_k \left|\mu_{j0}^k\right|^2$ with ΔE_j and μ_{j0}^k being the jth excitation energy and its associated transition dipole moment in the spatial direction k. Figure S4 shows the obtained oscillator strength distributions where the blue solid line shows the RT-TDDFT result and the red lines show the LR-TDDFT results. We find that the main peak positions of RT-TDDFT spectra are consistent with those of the corresponding LR-TDDFT spectra.

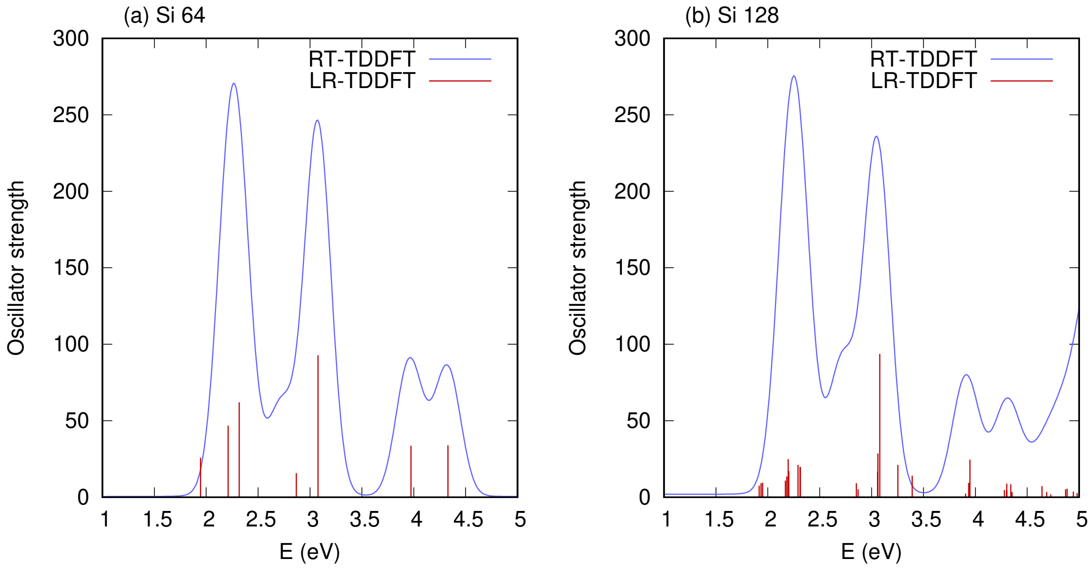

Figure S4: Oscillator strength distribution of silicon calculated using RT-TDDFT (blue solid line) and LR-TDDFT (red lines). Panel (a) shows the results of $2\times2\times2$ supercell (64 atoms) calculations, whereas panel (b) shows those of $2\times2\times4$ supercell (128 atoms) calculations.

References

(1) Jmol: an open-source Java viewer for chemical structures in 3D. http://www.jmol.org/

(2) VandeVondele, J.; Hutter, J. Gaussian basis sets for accurate calculations on molecular systems in gas and condensed phases. *J. Chem. Phys.* **2007**, *127*, 114105.

(3) Monkhorst, H. J.; Pack, J. D. Special points for Brillouin-zone integrations. *Phys. Rev. B* **1976**, *13*, 5188–5192.

(4) Hirata, S.; Head-Gordon, M. Time-dependent density functional theory within the Tamm-Dancoff approximation. *Chem. Phys. Lett.* **1999**, *314*, 291-299.

(5) Iannuzzi, M.; Chassaing, T.; Wallman, T.; Hutter, J. Ground and Excited State Density Functional Calculations With the Gaussian and Augmented-Plane-Wave Method. *Chimia* **2005**, *59 (7-8)*, 499.

(6) Perdew, J. P.; Zunger, A. Self-interaction correction to density-functional approximations for many-electron systems. *Phys. Rev. B* **1981**, *23*, 5048–5079.

(7) Goedecker, S.; Teter, M.; Hutter, J. Separable dual-space Gaussian pseudopotentials. *Phys. Rev. B* **1996**, *54*, 1703–1710.
